# MUSICAL THEATER AND MEANING MAKING IN A RETIREMENT COMMUNITY

**DOI:** 10.1093/geroni/igad104.0909

**Published:** 2023-12-21

**Authors:** James Powers, Robyn Stone

## Abstract

“I think I’ve had it up to here with pickleball…” Thus begins the first song in “Movin”, an original musical written, produced, and performed by members of a continuing care retirement community (CCRC), which addresses themes ranging from dementia, death and widowhood to finding community, purpose, and late-life love. The idea began in informal conversation between two former university colleagues, Philip Sloane (a geriatrician, GSA member, and former CCRC board member) and Hugh Tilson (a CCRC resident, aged 82) about describing the aging experience through song. Hugh gathered a group of fellow CCRC residents, none of whom had professional songwriting or theater experience but several of whom had musical backgrounds or an interest in theater. Meeting regularly over nearly two years, they envisioned, created, and then produced an original musical around the theme of “freshman year in a retirement community.” The final production included 9 cast members, 12 original songs developed by five lyricists and two composers, many production-related volunteers, and a narration to link the songs together. This symposium will describe the process, present and illustrate aging-related themes from the production, place the musical in the context of existing research about meaning-making and the arts in later life, and conclude with a discussion by a senior leader in a major senior housing organization. Presentation about development and production of the musical will include videos of rehearsals, songs from the production, and interviews with performers. Sponsored by the Humanities, Arts, and Cultural Gerontology Advisory Panel

